# 3-(3,4-Dimeth­oxy­phen­yl)-4-(2-meth­oxy­anilino)furan-2(5*H*)-one

**DOI:** 10.1107/S1600536811031266

**Published:** 2011-08-11

**Authors:** Chun-Lian Tian, Qijian Tian

**Affiliations:** a Key Laboratory of Hunan Forest Product and Chemical Industry Engineering, Jishou University, Zhangjiajie 427000, People’s Republic of China; b Key Laboratory of Plant Resources Conservation and Utilization of Hunan Province, Jishou University, Jishou 416000, People’s Republic of China

## Abstract

In the title compound, C_19_H_19_NO_5_, the furan­one unit makes a dihedral angle of 30.93 (6)° with the benzene ring and a dihedral angle of 9.51 (6)° with the aniline ring. In the crystal, inter­molecular C—H⋯O hydrogen bonds and C—H⋯π contacts link the mol­ecules into sheets. A weak intramolecular hydrogen bond is also observed.

## Related literature

For the biological activity of furan-2(5*H*)-one derivatives, see: Xiao, He *et al.* (2011 ▶). For related structures, see: Xiao *et al.* (2010 ▶); Xiao, Peng *et al.* (2011 ▶).
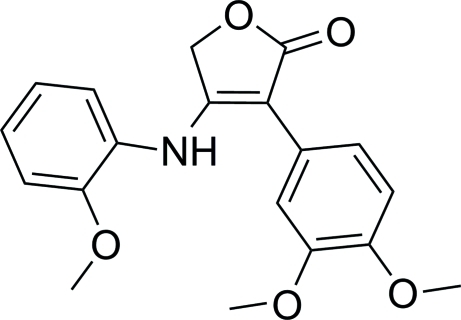


## Experimental

### 

#### Crystal data

C_19_H_19_NO_5_
*M* *_r_* = 341.35
Orthorhombic, 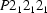

*a* = 7.4932 (5) Å
*b* = 11.5862 (7) Å
*c* = 18.5744 (12) Å
*V* = 1612.59 (18) Å^3^
*Z* = 4
Mo *K*α radiation
μ = 0.10 mm^−1^
*T* = 298 K
0.30 × 0.30 × 0.20 mm

#### Data collection

Bruker SMART APEX CCD diffractometer
Absorption correction: multi-scan (*SADABS*; Sheldrick, 1996 ▶) *T*_min_ = 0.970, *T*_max_ = 0.980
10601 measured reflections
3940 independent reflections
3732 reflections with *I* > 2σ(*I*)
*R*_int_ = 0.046

#### Refinement

*R*[*F* ^2^ > 2σ(*F* ^2^)] = 0.048
*wR*(*F* ^2^) = 0.121
*S* = 1.09
3940 reflections
233 parameters
H atoms treated by a mixture of independent and constrained refinement
Δρ_max_ = 0.24 e Å^−3^
Δρ_min_ = −0.34 e Å^−3^

### 

Data collection: *SMART* (Bruker, 2007 ▶); cell refinement: *SAINT* (Bruker, 2007 ▶); data reduction: *SAINT*; program(s) used to solve structure: *SHELXS97* (Sheldrick, 2008 ▶); program(s) used to refine structure: *SHELXL97* (Sheldrick, 2008 ▶); molecular graphics: *SHELXTL* (Sheldrick, 2008 ▶); software used to prepare material for publication: *SHELXL97*.

## Supplementary Material

Crystal structure: contains datablock(s) global, I. DOI: 10.1107/S1600536811031266/pv2437sup1.cif

Structure factors: contains datablock(s) I. DOI: 10.1107/S1600536811031266/pv2437Isup2.hkl

Supplementary material file. DOI: 10.1107/S1600536811031266/pv2437Isup3.cml

Additional supplementary materials:  crystallographic information; 3D view; checkCIF report

## Figures and Tables

**Table 1:** Hydrogen-bond geometry (Å, °) *Cg* is the centroid of the C11–C16 ring.

*D*—H⋯*A*	*D*—H	H⋯*A*	*D*⋯*A*	*D*—H⋯*A*
C2—H2⋯O1	0.93	2.43	3.006 (2)	121
C17—H17*B*⋯O1^i^	0.96	2.58	3.428 (2)	147
C17—H17*C*⋯*Cg*^ii^	0.96	2.94	3.847 (2)	158
C19—H19*C*⋯*Cg*^iii^	0.96	2.77	3.695 (2)	162

Symmetry codes: (i) 
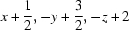
; (ii) 
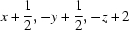
; (iii) 
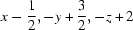
.

**Fig. 1.**
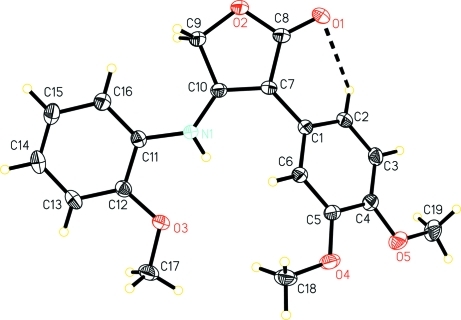
Molecular structure of the title compound, showing the atom-numbering scheme. Displacement ellipsoids are drawn at the 30% probability level.

**Fig. 2.**
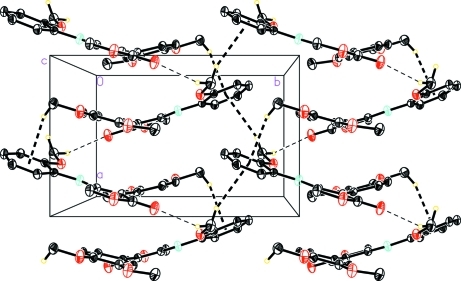
Packing diagram of the title compound viewed along the c axis, showing intermolecular C—H···O hydrogen bonds and C—H···π contacts. For the sake of clarity, the H atoms not involved in the hydrogen bonds have been omitted.

## supplementary crystallographic information

### Comment

Furan-2(5*H*)-one is a part of many natural and synthetic compounds, which possess useful biological activities (Xiao, He *et al.*, 2011). Herein, we reported the crystal structure of the title compound which is a derivatives of furan-2(5*H*)-one.

In the title molecule (Fig. 1), the bond distance C7—C10 (1.358 (2) Å) is indicative of a double bond which is consistent with the corresponding bond distances in the analogues of the title compound reported recently (Xiao *et al.*, 2010; Xiao, Peng *et al.*, 2011). The bond distance C10—N1 (1.359 (2) Å) is shorter than the standard C—N single bond (1.48 Å), but longer than a C═N double bond (1.28 Å). This clearly indicated that a *p* orbital of N1 is conjugated with the π molecular orbital of C7═C10 double bond. Moreover, 3,4-dimethoxybenzene moiety and aniline ring form dihedral angles of 30.93 (6) ° and 9.51 (6) ° with the central furan-2(5*H*)-one ring, respectively.

The molecules of the title compound are connected by intermolecular C17—H17B···O1 hydrogen bonds and C—H···π interactions to generate two-dimensional sheets of molecules (Tab. 1 & Fig. 2).

### Experimental

To a mixture of 2-methoxyaniline (147 mg, 1.2 mmol) and *p*-toluene sulphonic acid (6.8 mg, 0.04 mmol) waqs added 3-(3,4-dimethoxyphenyl)-4-hydroxyfuran-2(5*H*)-one (236 mg, 1 mmol) which was prepared according to the procedure described earlier (Xiao, He *et al.*, 2011). The mixture was heated to 370 K for 20 min. and toluene (12 ml) was then added and refluxed for 8 h. After toluene was removed under reduced pressure, the residue was purified by column chromatography on silica gel, eluting with EtOAc/petroleum ether (1:1). The crystals of the title compound were grown from EtOAc/petroleum ether (1:1) at room temperature by slow evaporation.

### Refinement

The H atom bonded to N1 was located from a difference Fourier map and was allowed to refine. The rest of the H atoms were placed in geometrically idealized positions and constrained to ride on their parent atoms with C—H = 0.9 , 0.97 and 0.96 Å for aromatic, methylene and methyl type H atoms, respectively, with *U*_iso_(H) = 1.5 times *U*_eq_(C) for methyl H-atoms and 1.2 times *U*_eq_(C) for the rest of the H-atoms. An absolute structure was not established in this analysis.

### Crystal data

**Table:**

C_19_H_19_NO_5_	*F*(000) = 720
*M**_r_* = 341.35	*D*_x_ = 1.406 Mg m^−^^3^
Orthorhombic, *P*2_1_2_1_2_1_	Mo *K*α radiation, λ = 0.71073 Å
Hall symbol: P 2ac 2ab	Cell parameters from 3629 reflections
*a* = 7.4932 (5) Å	θ = 2.4–27.8°
*b* = 11.5862 (7) Å	µ = 0.10 mm^−^^1^
*c* = 18.5744 (12) Å	*T* = 298 K
*V* = 1612.59 (18) Å^3^	Block, colorless
*Z* = 4	0.30 × 0.30 × 0.20 mm

### Data collection

**Table:**

Bruker SMART APEX CCD diffractometer	3940 independent reflections
Radiation source: fine-focus sealed tube	3732 reflections with *I* > 2σ(*I*)
graphite	*R*_int_ = 0.046
φ and ω scans	θ_max_ = 28.3°, θ_min_ = 2.1°
Absorption correction: multi-scan (*SADABS*; Sheldrick, 1996)	*h* = −9→9
*T*_min_ = 0.970, *T*_max_ = 0.980	*k* = −14→15
10601 measured reflections	*l* = −19→24

### Refinement

**Table:**

Refinement on *F*^2^	Primary atom site location: structure-invariant direct methods
Least-squares matrix: full	Secondary atom site location: difference Fourier map
*R*[*F*^2^ > 2σ(*F*^2^)] = 0.048	Hydrogen site location: inferred from neighbouring sites
*wR*(*F*^2^) = 0.121	H atoms treated by a mixture of independent and constrained refinement
*S* = 1.09	*w* = 1/[σ^2^(*F*_o_^2^) + (0.0689*P*)^2^ + 0.1364*P*] where *P* = (*F*_o_^2^ + 2*F*_c_^2^)/3
3940 reflections	(Δ/σ)_max_ = 0.001
233 parameters	Δρ_max_ = 0.24 e Å^−^^3^
0 restraints	Δρ_min_ = −0.34 e Å^−^^3^

### Special details

**Table:**

Geometry. All e.s.d.'s (except the e.s.d. in the dihedral angle between two l.s. planes) are estimated using the full covariance matrix. The cell e.s.d.'s are taken into account individually in the estimation of e.s.d.'s in distances, angles and torsion angles; correlations between e.s.d.'s in cell parameters are only used when they are defined by crystal symmetry. An approximate (isotropic) treatment of cell e.s.d.'s is used for estimating e.s.d.'s involving l.s. planes.
Refinement. Refinement of *F*^2^ against ALL reflections. The weighted *R*-factor *wR* and goodness of fit *S* are based on *F*^2^, conventional *R*-factors *R* are based on *F*, with *F* set to zero for negative *F*^2^. The threshold expression of *F*^2^ > σ(*F*^2^) is used only for calculating *R*-factors(gt) *etc*. and is not relevant to the choice of reflections for refinement. *R*-factors based on *F*^2^ are statistically about twice as large as those based on *F*, and *R*- factors based on ALL data will be even larger.

### Fractional atomic coordinates and isotropic or equivalent isotropic displacement parameters (Å^2^)

**Table:**

	*x*	*y*	*z*	*U*_iso_*/*U*_eq_	
C1	0.1004 (2)	0.78788 (13)	0.96299 (8)	0.0298 (3)	
C2	0.1438 (2)	0.90439 (13)	0.96587 (9)	0.0357 (3)	
H2	0.1580	0.9453	0.9232	0.043*	
C3	0.1665 (2)	0.96090 (14)	1.03105 (10)	0.0386 (4)	
H3	0.1968	1.0387	1.0314	0.046*	
C4	0.1447 (2)	0.90327 (15)	1.09529 (9)	0.0380 (4)	
C5	0.0948 (2)	0.78615 (14)	1.09393 (9)	0.0354 (3)	
C6	0.0722 (2)	0.73015 (13)	1.02868 (8)	0.0325 (3)	
H6	0.0379	0.6530	1.0283	0.039*	
C7	0.0929 (2)	0.72736 (13)	0.89352 (8)	0.0306 (3)	
C8	0.0519 (2)	0.78617 (14)	0.82608 (9)	0.0375 (4)	
C9	0.1286 (3)	0.60193 (14)	0.79642 (8)	0.0410 (4)	
H9A	0.2447	0.5807	0.7774	0.049*	
H9B	0.0429	0.5428	0.7833	0.049*	
C10	0.1364 (2)	0.61684 (14)	0.87662 (8)	0.0313 (3)	
C11	0.2423 (2)	0.41733 (13)	0.91057 (9)	0.0327 (3)	
C12	0.3074 (2)	0.35932 (13)	0.97200 (9)	0.0337 (3)	
C13	0.3749 (2)	0.24856 (14)	0.96556 (10)	0.0423 (4)	
H13	0.4209	0.2110	1.0057	0.051*	
C14	0.3739 (3)	0.19349 (15)	0.89930 (12)	0.0478 (4)	
H14	0.4209	0.1195	0.8951	0.057*	
C15	0.3044 (3)	0.24702 (15)	0.84022 (11)	0.0452 (4)	
H15	0.3022	0.2087	0.7962	0.054*	
C16	0.2368 (2)	0.35889 (15)	0.84532 (9)	0.0401 (4)	
H16	0.1880	0.3944	0.8050	0.048*	
C17	0.3697 (3)	0.37008 (17)	1.09825 (10)	0.0503 (5)	
H17A	0.2980	0.3053	1.1125	0.075*	
H17B	0.3706	0.4264	1.1362	0.075*	
H17C	0.4895	0.3447	1.0890	0.075*	
C18	0.0450 (3)	0.61586 (16)	1.16219 (10)	0.0514 (5)	
H18A	−0.0672	0.5970	1.1402	0.077*	
H18B	0.0447	0.5905	1.2114	0.077*	
H18C	0.1398	0.5780	1.1366	0.077*	
C19	0.2239 (3)	1.06783 (16)	1.16409 (12)	0.0539 (5)	
H19A	0.3331	1.0760	1.1375	0.081*	
H19B	0.2431	1.0908	1.2131	0.081*	
H19C	0.1336	1.1158	1.1429	0.081*	
H1	0.210 (3)	0.5550 (18)	0.9629 (12)	0.046 (6)*	
N1	0.1861 (2)	0.53176 (12)	0.92278 (7)	0.0360 (3)	
O1	0.0054 (2)	0.88447 (10)	0.81447 (7)	0.0520 (4)	
O2	0.0742 (2)	0.71219 (10)	0.76969 (6)	0.0484 (3)	
O3	0.29681 (17)	0.42052 (10)	1.03438 (6)	0.0398 (3)	
O4	0.0713 (2)	0.73683 (11)	1.15974 (7)	0.0529 (4)	
O5	0.1675 (2)	0.95037 (11)	1.16213 (7)	0.0532 (4)	

### Atomic displacement parameters (Å^2^)

**Table:**

	*U*^11^	*U*^22^	*U*^33^	*U*^12^	*U*^13^	*U*^23^
C1	0.0314 (7)	0.0298 (7)	0.0282 (7)	0.0035 (5)	0.0000 (5)	0.0007 (6)
C2	0.0432 (8)	0.0308 (7)	0.0331 (7)	0.0034 (6)	0.0009 (7)	0.0070 (6)
C3	0.0450 (9)	0.0253 (7)	0.0456 (9)	0.0010 (6)	−0.0016 (8)	−0.0022 (6)
C4	0.0433 (9)	0.0358 (8)	0.0349 (8)	0.0017 (7)	−0.0039 (7)	−0.0054 (7)
C5	0.0440 (9)	0.0337 (7)	0.0284 (7)	−0.0004 (7)	−0.0007 (6)	0.0006 (6)
C6	0.0395 (8)	0.0287 (7)	0.0292 (7)	−0.0005 (6)	−0.0007 (6)	0.0010 (6)
C7	0.0365 (8)	0.0314 (7)	0.0240 (7)	0.0013 (6)	−0.0001 (5)	0.0044 (5)
C8	0.0483 (9)	0.0369 (8)	0.0272 (7)	0.0023 (7)	−0.0013 (6)	0.0050 (6)
C9	0.0625 (10)	0.0341 (8)	0.0264 (7)	0.0028 (8)	−0.0008 (7)	0.0022 (6)
C10	0.0357 (7)	0.0334 (7)	0.0248 (7)	−0.0007 (6)	−0.0003 (5)	0.0020 (6)
C11	0.0332 (7)	0.0292 (7)	0.0359 (8)	0.0002 (6)	0.0017 (6)	0.0016 (6)
C12	0.0348 (7)	0.0299 (7)	0.0365 (8)	−0.0035 (6)	−0.0008 (6)	0.0037 (6)
C13	0.0447 (9)	0.0294 (8)	0.0528 (10)	0.0015 (7)	−0.0034 (8)	0.0102 (7)
C14	0.0501 (10)	0.0269 (7)	0.0664 (12)	0.0017 (7)	0.0047 (9)	−0.0025 (8)
C15	0.0519 (10)	0.0340 (8)	0.0496 (10)	−0.0034 (8)	0.0080 (8)	−0.0089 (8)
C16	0.0472 (9)	0.0360 (8)	0.0369 (9)	−0.0010 (7)	−0.0001 (7)	−0.0015 (7)
C17	0.0676 (12)	0.0442 (10)	0.0392 (9)	−0.0006 (9)	−0.0172 (9)	0.0095 (7)
C18	0.0718 (13)	0.0466 (10)	0.0357 (9)	−0.0087 (9)	−0.0026 (9)	0.0116 (8)
C19	0.0584 (12)	0.0448 (10)	0.0586 (12)	−0.0023 (9)	−0.0102 (10)	−0.0203 (9)
N1	0.0534 (9)	0.0302 (6)	0.0245 (6)	0.0080 (6)	−0.0046 (6)	−0.0010 (5)
O1	0.0830 (10)	0.0373 (6)	0.0358 (7)	0.0139 (7)	−0.0052 (6)	0.0104 (5)
O2	0.0808 (10)	0.0388 (6)	0.0256 (5)	0.0069 (6)	−0.0025 (6)	0.0060 (5)
O3	0.0528 (7)	0.0350 (6)	0.0315 (6)	0.0025 (5)	−0.0074 (5)	0.0055 (4)
O4	0.0895 (10)	0.0430 (7)	0.0262 (6)	−0.0069 (7)	−0.0014 (6)	−0.0004 (5)
O5	0.0796 (10)	0.0422 (7)	0.0378 (7)	−0.0057 (6)	−0.0079 (7)	−0.0112 (5)

### Geometric parameters (Å, °)

**Table:**

C1—C2	1.390 (2)	C11—C12	1.411 (2)
C1—C6	1.407 (2)	C12—O3	1.3607 (19)
C1—C7	1.470 (2)	C12—C13	1.384 (2)
C2—C3	1.387 (2)	C13—C14	1.386 (3)
C2—H2	0.9300	C13—H13	0.9300
C3—C4	1.377 (2)	C14—C15	1.364 (3)
C3—H3	0.9300	C14—H14	0.9300
C4—O5	1.367 (2)	C15—C16	1.395 (2)
C4—C5	1.408 (2)	C15—H15	0.9300
C5—O4	1.361 (2)	C16—H16	0.9300
C5—C6	1.385 (2)	C17—O3	1.431 (2)
C6—H6	0.9300	C17—H17A	0.9600
C7—C10	1.358 (2)	C17—H17B	0.9600
C7—C8	1.459 (2)	C17—H17C	0.9600
C8—O1	1.210 (2)	C18—O4	1.416 (2)
C8—O2	1.364 (2)	C18—H18A	0.9600
C9—O2	1.4299 (19)	C18—H18B	0.9600
C9—C10	1.501 (2)	C18—H18C	0.9600
C9—H9A	0.9700	C19—O5	1.425 (2)
C9—H9B	0.9700	C19—H19A	0.9600
C10—N1	1.359 (2)	C19—H19B	0.9600
C11—C16	1.389 (2)	C19—H19C	0.9600
C11—N1	1.4093 (19)	N1—H1	0.81 (2)
			
C2—C1—C6	117.60 (13)	C13—C12—C11	119.85 (15)
C2—C1—C7	120.42 (13)	C12—C13—C14	120.10 (16)
C6—C1—C7	121.92 (13)	C12—C13—H13	120.0
C3—C2—C1	121.40 (14)	C14—C13—H13	120.0
C3—C2—H2	119.3	C15—C14—C13	120.47 (16)
C1—C2—H2	119.3	C15—C14—H14	119.8
C4—C3—C2	120.86 (14)	C13—C14—H14	119.8
C4—C3—H3	119.6	C14—C15—C16	120.44 (17)
C2—C3—H3	119.6	C14—C15—H15	119.8
O5—C4—C3	125.35 (15)	C16—C15—H15	119.8
O5—C4—C5	115.74 (15)	C11—C16—C15	120.10 (16)
C3—C4—C5	118.91 (14)	C11—C16—H16	119.9
O4—C5—C6	125.00 (14)	C15—C16—H16	119.9
O4—C5—C4	115.02 (14)	O3—C17—H17A	109.5
C6—C5—C4	119.98 (14)	O3—C17—H17B	109.5
C5—C6—C1	121.18 (14)	H17A—C17—H17B	109.5
C5—C6—H6	119.4	O3—C17—H17C	109.5
C1—C6—H6	119.4	H17A—C17—H17C	109.5
C10—C7—C8	107.00 (13)	H17B—C17—H17C	109.5
C10—C7—C1	130.07 (13)	O4—C18—H18A	109.5
C8—C7—C1	122.63 (14)	O4—C18—H18B	109.5
O1—C8—O2	119.34 (14)	H18A—C18—H18B	109.5
O1—C8—C7	130.77 (16)	O4—C18—H18C	109.5
O2—C8—C7	109.89 (13)	H18A—C18—H18C	109.5
O2—C9—C10	104.64 (13)	H18B—C18—H18C	109.5
O2—C9—H9A	110.8	O5—C19—H19A	109.5
C10—C9—H9A	110.8	O5—C19—H19B	109.5
O2—C9—H9B	110.8	H19A—C19—H19B	109.5
C10—C9—H9B	110.8	O5—C19—H19C	109.5
H9A—C9—H9B	108.9	H19A—C19—H19C	109.5
C7—C10—N1	127.16 (14)	H19B—C19—H19C	109.5
C7—C10—C9	109.19 (13)	C10—N1—C11	131.53 (14)
N1—C10—C9	123.60 (15)	C10—N1—H1	113.5 (15)
C16—C11—N1	126.17 (15)	C11—N1—H1	113.1 (15)
C16—C11—C12	118.92 (14)	C8—O2—C9	109.26 (12)
N1—C11—C12	114.91 (14)	C12—O3—C17	118.09 (13)
O3—C12—C13	125.31 (14)	C5—O4—C18	117.54 (14)
O3—C12—C11	114.83 (13)	C4—O5—C19	116.20 (15)
			
C6—C1—C2—C3	2.8 (2)	O2—C9—C10—N1	−178.82 (16)
C7—C1—C2—C3	−174.47 (14)	C16—C11—C12—O3	−175.81 (14)
C1—C2—C3—C4	−0.7 (2)	N1—C11—C12—O3	3.5 (2)
C2—C3—C4—O5	178.57 (16)	C16—C11—C12—C13	4.0 (2)
C2—C3—C4—C5	−1.4 (2)	N1—C11—C12—C13	−176.63 (15)
O5—C4—C5—O4	1.8 (2)	O3—C12—C13—C14	178.05 (16)
C3—C4—C5—O4	−178.21 (16)	C11—C12—C13—C14	−1.8 (3)
O5—C4—C5—C6	−178.62 (15)	C12—C13—C14—C15	−0.9 (3)
C3—C4—C5—C6	1.4 (2)	C13—C14—C15—C16	1.3 (3)
O4—C5—C6—C1	−179.67 (16)	N1—C11—C16—C15	177.11 (17)
C4—C5—C6—C1	0.8 (2)	C12—C11—C16—C15	−3.6 (2)
C2—C1—C6—C5	−2.8 (2)	C14—C15—C16—C11	1.0 (3)
C7—C1—C6—C5	174.39 (15)	C7—C10—N1—C11	−176.24 (17)
C2—C1—C7—C10	145.33 (17)	C9—C10—N1—C11	1.2 (3)
C6—C1—C7—C10	−31.8 (3)	C16—C11—N1—C10	−9.3 (3)
C2—C1—C7—C8	−27.5 (2)	C12—C11—N1—C10	171.42 (17)
C6—C1—C7—C8	155.39 (16)	O1—C8—O2—C9	−179.88 (18)
C10—C7—C8—O1	179.3 (2)	C7—C8—O2—C9	0.6 (2)
C1—C7—C8—O1	−6.4 (3)	C10—C9—O2—C8	0.2 (2)
C10—C7—C8—O2	−1.2 (2)	C13—C12—O3—C17	3.7 (2)
C1—C7—C8—O2	173.04 (15)	C11—C12—O3—C17	−176.45 (15)
C8—C7—C10—N1	179.05 (16)	C6—C5—O4—C18	8.8 (3)
C1—C7—C10—N1	5.4 (3)	C4—C5—O4—C18	−171.59 (17)
C8—C7—C10—C9	1.34 (19)	C3—C4—O5—C19	−2.1 (3)
C1—C7—C10—C9	−172.34 (16)	C5—C4—O5—C19	177.94 (16)
O2—C9—C10—C7	−1.0 (2)		

### Hydrogen-bond geometry (Å, °)

**Table:**

Cg is the centroid of the C11–C16 ring.

**Table:**

*D*—H···*A*	*D*—H	H···*A*	*D*···*A*	*D*—H···*A*
C2—H2···O1	0.93	2.43	3.006 (2)	121.
C17—H17B···O1^i^	0.96	2.58	3.428 (2)	147.
C17—H17C···Cg^ii^	0.96	2.94	3.847 (2)	158
C19—H19C···Cg^iii^	0.96	2.77	3.695 (2)	162

Symmetry codes: (i) *x*+1/2, −*y*+3/2, −*z*+2; (ii) *x*+1/2, −*y*+1/2, −*z*+2; (iii) *x*−1/2, −*y*+3/2, −*z*+2.
